# *APOE* genotype and brain amyloid are associated with changes in the plasma proteome in elderly subjects without dementia

**DOI:** 10.1002/acn3.52250

**Published:** 2024-12-17

**Authors:** Sarah M. Philippi, Kailash BP, Towfique Raj, Joseph M. Castellano

**Affiliations:** ^1^ Nash Family Department of Neuroscience, Department of Neurology, Friedman Brain Institute Icahn School of Medicine at Mount Sinai New York New York USA; ^2^ Ronald M. Loeb Center for Alzheimer's Disease Icahn School of Medicine at Mount Sinai New York New York USA; ^3^ Graduate School of Biomedical Sciences Icahn School of Medicine at Mount Sinai New York New York USA; ^4^ Black Family Stem Cell Institute Icahn School of Medicine at Mount Sinai New York New York USA; ^5^ Department of Genetics and Genomic Sciences, Icahn Institute for Data Science and Genomic Technology Icahn School of Medicine at Mount Sinai New York New York USA

## Abstract

**Objective:**

Recent work has bolstered the possibility that peripheral changes may be relevant to Alzheimer's disease pathogenesis in the brain. While age‐associated blood‐borne proteins have been targeted to restore function to the aged brain, it remains unclear whether other dysfunctional systemic states can be exploited for similar benefits. Here, we investigate whether *APOE* allelic variation or presence of brain amyloid are associated with plasma proteomic changes and the molecular processes associated with these changes.

**Methods:**

Using the SOMAscan assay, we measured 1305 plasma proteins from 53 homozygous, *APOE3* and *APOE4* subjects without dementia. We investigated the relationship of either the *APOE‐ε4* allele or amyloid positivity with plasma proteome changes by linear mixed effects modeling and ontology‐based pathway and module–trait correlation analyses.

**Results:**

*APOE4* is associated with plasma protein differences linked to atherosclerosis, tyrosine kinase activity, cholesterol transport, extracellular matrix, and synaptogenesis pathways. Independent of *APOE4*, we found that subjects likely harboring brain amyloid exhibit plasma proteome signatures associated with AD‐linked pathways, including neurovascular dysfunction.

**Interpretation:**

Our results indicate that *APOE4* status or presence of brain amyloid are associated with plasma proteomic shifts prior to the onset of symptoms, suggesting that systemic pathways in certain risk contexts may be plausible targets for disease modification.

## Introduction

Alzheimer's disease (AD) is a devastating neurodegenerative disease for which aging serves as the strongest risk factor. The development of AD is associated with several hallmark pathologies, including the accumulation of amyloid‐β into plaques, which can be detected in living subjects using a variety of reliable biomarkers.^1^ As the global population ages, more effective treatments are needed to substantially change the course of disease. One intriguing direction has been to explore how aging acts as a risk factor for AD, particularly through characterization of blood–CNS communication pathways that have been shown to be perturbed in mice and humans.^2^, ^3^, ^4^, ^5^, ^6^, ^7^, ^8^ These studies support the idea that age‐associated changes in the plasma proteome can be harnessed to revitalize age‐sensitive organ systems.^2^ Our group and others have demonstrated the sufficiency of youth‐associated blood‐borne proteins, via parabiosis or through plasma transfer, to revitalize the aged mouse brain and restore hippocampal function.^3^ Intriguingly, we found that human plasma proteins were sufficient to recapitulate these phenotypes in aged mice.^3^ While these studies demonstrate that brain function can be modified by aging‐associated blood‐borne proteins, it remains unclear whether pathological processes within the AD brain alter the systemic environment or whether genetic risk variants modulate the composition of the blood proteome to affect brain health.

The strongest genetic risk factor for sporadic, late‐onset AD is possession of the *APOE‐ε4* allele.^9^ *APOE*‐*ε4*‐carriers exhibit earlier onset of β‐amyloidosis relative to other alleles,^10^ and animal studies suggest that earlier onset of deposition can be attributed to impaired ability of apoE4 to clear soluble Aβ early in life.^11^ Other deleterious phenotypes have been attributed to *APOE4* in mice, including altered synaptic integrity and increased neuroinflammation, some of which may be amyloid‐independent.^12^ In light of work tying the systemic environment to CNS health,^2^ the relative contribution of peripheral and CNS apoE pools in mediating risk is poorly understood. Recent studies highlight the role of hepatic apoE expression in regulating synaptic integrity in mice with humanized *APOE4* livers,^13^, ^14^ while ablation of hepatic apoE on its own failed to alter cerebral amyloid deposition.^15^ In cases of symptomatic disease or advancing age, specific proteins appear altered in cerebrospinal fluid (CSF)^16^, ^17^, ^18^ and plasma of *APOE4‐*carriers.^18^, ^19^, ^20^, ^21^, ^22^, ^23^ It is thus likely that peripheral apoE regulates the systemic environment in an allele‐dependent manner, though characterization of how *APOE4* affects the plasma proteome in elderly subjects without dementia is lacking.

While recent work has examined differential protein abundance in tissue^24^ or CSF^16^, ^17^ as readouts of brain health, sensitive methods are needed to probe changes conferred by AD risk variants and in a manner that minimizes discomfort to subjects. A large body of work has explored minimally invasive peripheral markers, namely blood‐based markers associated with AD signatures like amyloid‐β deposition,^25^ that can be measured before symptomatic disease onset. Conventional methods to measure plasma proteins suffer from limited throughput and an overall lack of suitable immunoassays for specific proteins. To overcome these issues, we used the SOMAscan assay, which utilizes slow off‐rate modified aptamers (SOMAmers) to sensitively provide relative quantification for many human plasma proteins^19^ in a single assay, making it well‐suited for exploratory studies. We performed plasma proteomic profiling using SOMAscan to measure 1305 proteins in 53 elderly subjects without dementia homozygous for *APOE3* or *APOE4*, which facilitated identification of *APOE*‐associated or amyloid‐associated plasma proteins. Ontology‐based over‐representation analyses revealed that expression of *APOE4* is associated with plasma protein perturbations linked to pathways involving atherosclerosis, ECM, and neuronal and synaptic function. In contrast, presence of brain amyloid was associated with plasma protein changes linked to immune and blood–brain barrier function, myelination, and pyroptosis signaling. Our findings, coupled with accumulating evidence that blood‐borne proteins modulate CNS health,^2^ support the concept that disruption present within the systemic environment in these contexts may represent putative targets for clinical intervention.

## Methods

### Plasma collection

Samples were provided as de‐identified samples from the Charles F. and Joanne Knight Alzheimer Disease Research Center (ADRC) at Washington University School of Medicine, and our study received an IRB human research exempt determination from Icahn School of Medicine at Mount Sinai. Requests for Knight ADRC data or biospecimens are directed to knightadrc.wustl.edu. Following recruitment, Knight ADRC determined *APOE* allelic status by genotyping rs7412 and rs429358 with Taqman genotyping technology.^26^ The well‐characterized Clinical Dementia Rating (CDR) scale was used, and scores were assigned following neurological examination by Knight ADRC according to established protocols.^27^ The Mini‐Mental State Examination (MMSE) was used to confirm subjects did not have dementia.^28^ Whole blood samples were drawn from *N* = 53 fasted subjects into 10 mL syringes pre‐coated with 0.5 M EDTA and transferred to 15 mL polypropylene tubes containing 120 μL of 0.5 M EDTA. Samples were kept on ice until centrifugation (<2 h) at 2000 *g* × 17 min, allowing separation of plasma from the cellular fraction. Plasma was pooled and aliquoted into new polypropylene tubes (300–500 μL) prior to storage at −80°C, as previously described.^29^, ^30^ Brain amyloid status was assigned using a threshold based on previous work for CSF Aβ42 < 1098 pg/mL (amyloid‐positive) or CSF Aβ42 > 1098 pg/mL (amyloid‐negative) determined by the Elecsys CSF Aβ42 assay.^31^ Male and female subjects with CDR = 0 homozygous for either *APOE3* or *APOE4* were selected for plasma proteomic measurements. No samples from *APOE2* subjects were included in the study. Plasma samples that corresponded to CSF collection dates were analyzed.

### SOMAscan assay

Proteomic profiling of 1305 SOMAmers was performed using 1.3 k SOMAscan Assay^32^ at Genome Technology Access Center (WashU), a fee‐for‐service and SomaLogic authorized site. The SOMAscan plate is designed to include buffer wells and SomaLogic quality control and calibrator samples, all of which were run in duplicate. At least 50% of SOMAmer reagents had a coefficient of variation less than 0.1 and 95% had a CV below 0.2. With the exception of 12 hybridization elution controls removed prior to downstream analyses, no proteins were excluded in final analyses. Data were reported as SOMAmer reagent abundance in relative fluorescence units (RFU) with reagent abundance indicating protein concentration within the sample. Raw data were processed for hybridization normalization to adjust for individual sample variance. Median signal normalization was then performed to adjust sample‐to‐sample differences in RFU brightness. Calibration normalization was applied to all samples in the plate to remove variance due to assay run according to manufacturer recommendations.

### Covariate analysis

The variancePartition^33^ package in R (version 4.1.2) was used to calculate the proportion of variance in protein abundance explained by *APOE* genotype, amyloid status, age, sex, and SOMAscan subarray. The SOMAscan subarray was included to account for technical variability in protein abundance in the assay. The subarray is a variable that takes into account sample location (row). The variancePartition package uses linear mixed model‐based assessment to quantify the source of variation attributed to each variable and guide covariate selection for final analyses. The “canCorPairs” function was used to further quantify and interpret covariate drivers of variance. To examine *APOE4*‐associated proteins, we used a linear mixed model of the following form: protein ~ APOE_status + sex + age + (1|Subarray) and, for amyloid‐associated proteins, we used protein ~ Amyloid_status + sex + age + (1|Subarray).

### Differential protein abundance analysis

Differential expression for repeated measures (Dream)^34^ R function in the variancePartition package was used to test protein abundance associations by *APOE* genotype or brain amyloid status. When testing differential protein abundance by *APOE* genotype, only amyloid‐positive *APOE3* and *APOE4* subjects were included, as amyloid‐negative *APOE4* subjects were not available for cohort selection. When evaluating effects of brain amyloid status (i.e., amyloid‐positive vs. amyloid‐negative) on protein abundance, *APOE4* subjects were excluded for the same reason. SOMAscan's 12 elution proteins included in the assay were removed, and the remaining 1305 proteins were analyzed. Proteins were ranked according to differential abundance using log_2_‐fold‐changes. Top proteins were represented using boxplots created using the R package “beeswarm” from the residualized protein outputs following Dream. Briefly, covariates for age, sex, and SOMAscan subarray were regressed out, while the effects of *APOE* genotype or amyloid status were re‐introduced.

### Differential protein abundance visualization

Volcano plots following analysis of protein levels were constructed using the R package ggplot2. Customization of these plots used the packages ggrepel, dplyr, and ggeasy. The top five proteins according to ‐log_10_(*P*‐value) were labeled for both upregulated and downregulated proteins. *Z*‐scored values were used for unsupervised hierarchical protein cluster analysis in Cluster 3.0 before heat map visualization using Java TreeView 1.0.13 based on *P*‐value score ranking of top 50 percent of altered proteins following differential protein abundance analysis.

### Pathway analyses

Gene ontology over‐representation analyses were performed using clusterProfiler^35^ package (v4.12.0) in R. Nominally significant *P‐*values (*P* < 0.05) from differential abundance were used as threshold for input lists for pathway discovery tools. This protein list was sorted and analyzed separately, with proteins classified as upregulated using direction of log_2_‐fold change calculated from Dream. Over‐representation analyses were carried out using “enrichGO” and visualized by dotplot for select pathways for GO categories (biological processes, molecular functions, and cellular components) with pathway *P*‐values adjusted for multiple comparisons using the Benjamini–Hochberg procedure (FDR < 0.05). GO terms of interest from respective dotplots were visualized further using “treeplot” function, and proteins from these terms were extracted and evaluated for gene‐network connectivity using “cnetplot.” The Database for Annotation, Visualization, and Integrated Discovery (DAVID; v2022q3) was used for complementary enrichment analysis using differentially abundant proteins from Dream against measured proteins. Enrichment analysis performed using Reactome pathway database through DAVID was performed on upregulated proteins, and significance was assessed by EASE scores, a more conservative Fisher's exact *P*‐value. Default settings in DAVID were used (maximum EASE score (*P*‐value) was 0.1 and count threshold was 2) with top 10 pathways (*P* < 0.05) visualized using fold enrichment values calculated by DAVID. Additional pathway enrichment analysis was carried out by IPA (Qiagen) using proteins differentially abundant at *P* < 0.05 from Dream divided according to direction of change, with upregulated proteins submitted separately from downregulated proteins using IPA knowledge base as reference background. The top 10 significant pathways, adjusted for multiple comparisons using the Benjamini–Hochberg procedure (FDR < 0.05), were visualized using GraphPad Prism v9. Protein identification analysis was performed after submission to IPA and after extracting protein classifications from the molecules tab. Plots were created using R package Circos.^36^

### STRING

Functional and physical protein associations were evaluated via STRING and represented as networks using the following settings: evidence‐based network edges were used for medium confidence interactions (0.400) with active interaction sources from “textmining” and “database” entries. Only queried proteins were used as interactors, and proteins with no interactions were not included.

### Weighted gene correlation network analysis

Scale‐free co‐expression networks were constructed from proteins using R package weighted gene correlation network analysis (WGCNA)^37^ to identify protein modules with coordinated expression patterns according to *APOE* genotype or amyloid status. An adjacency matrix was calculated using soft powers at which the scale‐free topology fit index reached 0.90 with mean connectivity near 0. The adjacency matrix was then transformed into a topological overlap matrix (TOM). We performed this analysis using default parameters for signed networks with exception for the following settings: soft threshold power = 14, minimum module size = 10, cutting height = 0.99, merge cut height = 0.25, and deepSplit = TRUE. All 1305 proteins from the SOMAscan assay were assigned to modules represented by distinct color identifiers. Proteins not meeting criteria for module assignment were collectively placed in the “grey” module. The resulting modules of co‐expressed proteins were then used to calculate module eigenproteins to determine how correlated proteins were to a particular module. Modules with *P* < 0.05 were included in downstream gene ontology analyses, and proteins were extracted to identify protein–protein interactions using nodes and edges generated by “exportNetworkToCytoscape” function and then visualized using the ggraph R package. Statistical analyses throughout were performed in R (version 4.1.2), and standard packages were used where indicated in respective sections.

## Results

### Variation in *APOE* genotype is associated with altered plasma proteome

**Figure 1:**
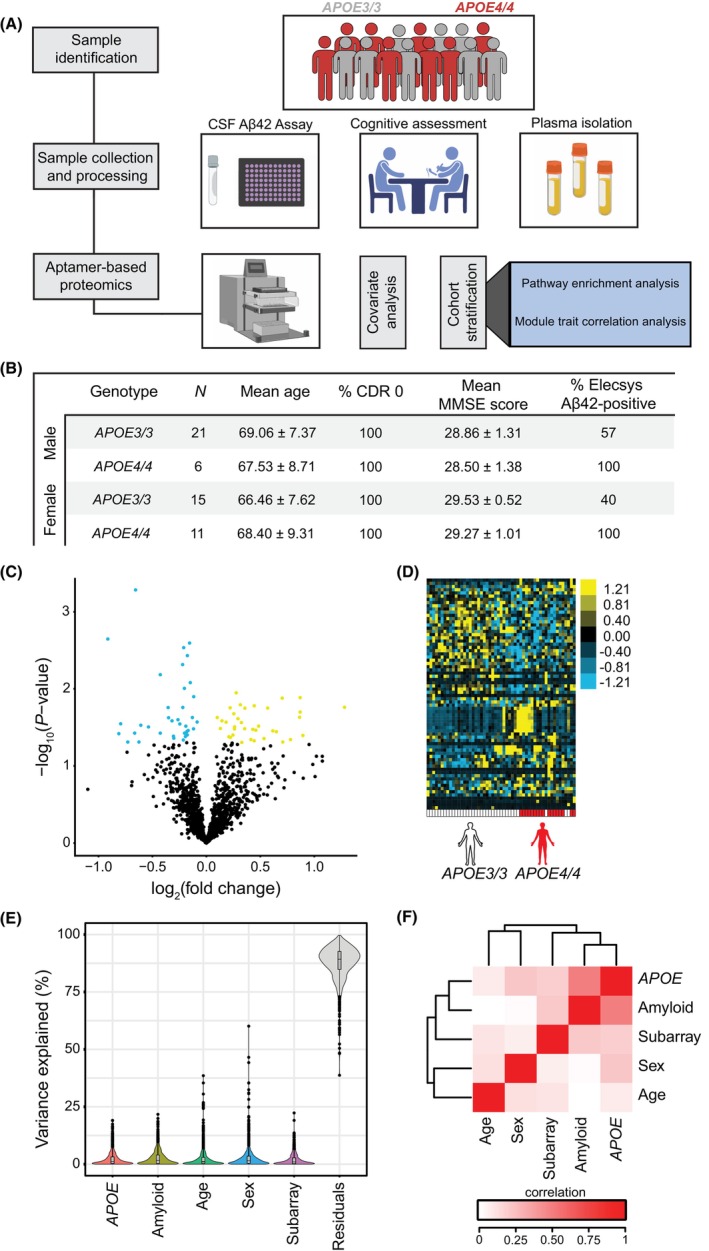
Variation in *APOE* alters the plasma proteome in elderly adults. (A) Schematic workflow describing sample selection and measurement of plasma samples from 53 *APOE3* and *APOE4* subjects from the Knight Alzheimer's Disease Research Center (WashU). CSF samples were collected from subjects by the Knight ADRC and run on Aβ42 Elecsys assays. Subjects underwent neurological examination and were classified using a Clinical Dementia Rating (CDR) score and Mini‐Mental State Examination (MMSE). Fresh blood samples and isolated plasma from *APOE3* and *APOE4* subjects were collected following established protocols. Relative abundance of 1305 proteins was measured using SOMAscan aptamer‐based profiling, followed by examination of *APOE* and amyloid‐associated effects on pathway enrichment and module‐trait relationships. (B) Male and female subjects with homozygous expression for *APOE3* or *APOE4* were evaluated. All participants had a CDR of 0 and MMSE score indicating the subjects did not have dementia. *APOE3* subjects were either amyloid‐positive or amyloid‐negative, whereas all *APOE4* subjects were amyloid‐positive, due to subject availability. (C) Volcano plot showing fold‐change of proteins (log_2_ scale) between *APOE3* and *APOE4* subjects and corresponding nominal *P*‐values (−log_10_ scale) with upregulated proteins highlighted in yellow and downregulated proteins highlighted in blue. (D) Heat map showing unsupervised hierarchical clustering of the differentially abundant proteins distinguished by *APOE* status. (E) Variance partitioning analysis for proteins showing individual covariate contributions to variance. (F) Covariate correlation analysis of biological and technical sources of variability. Degree to which these variables co‐vary is highlighted from white (no level of co‐variance) to red (high degree of co‐variance). Panel A was made using BioRender tools.

To begin to characterize changes in plasma protein levels due to variation of *APOE3* or *APOE4* alleles, we measured 1305 proteins in blood plasma from 36 *APOE3*/*3* and 17 *APOE4*/*4* elderly subjects (mean age = 68 years) (Fig. 1A,B), all of whom were without dementia, as assessed by both CDR and MMSE scores (Fig. 1B). We identified many plasma proteins that appeared to be up‐ or downregulated in those expressing *APOE4* relative to *APOE3* (Fig. 1C). Unsupervised hierarchical clustering revealed distinct separation according to *APOE* status in terms of plasma proteins (Fig. 1D). To examine sources of variation within the dataset, we performed variance partition analysis,^33^ which revealed that much of the variation is attributed to unknown biological or technical variables (Fig. 1E). To further decouple sources of variation, we also performed a canonical correlation analysis to identify the degree to which variables co‐vary. We identified strong correlation between *APOE* genotype and other variables, including brain amyloid status (Fig. 1F), arguing for subsequent cohort stratification by these variables (Fig. S1A–C). Thus, while *APOE4* appears to alter the plasma proteome relative to *APOE3* in elderly subjects, controlling for additional sources of biological variation may improve identification of individual protein‐level changes conferred by *APOE4*.

**Figure 2:**
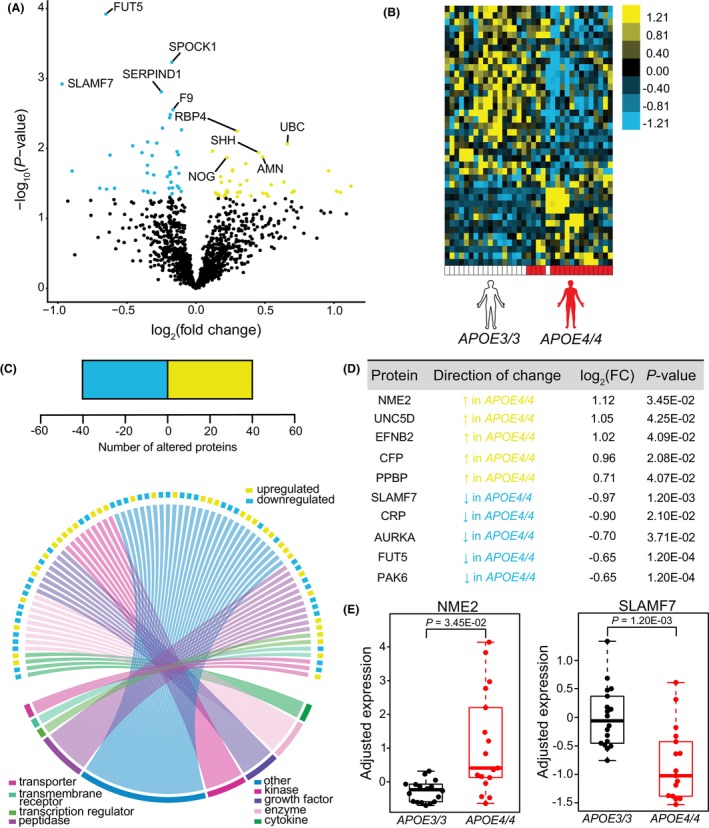
Plasma proteome features from amyloid‐positive *APOE3* and *APOE4* subjects without dementia. (A) Volcano plot showing the fold‐change of proteins (log_2_ scale) between *APOE3* and *APOE4* subjects and corresponding nominal *P*‐values (−log_10_ scale) with upregulated proteins highlighted in yellow and downregulated proteins highlighted in blue. (B) Heat map showing unsupervised hierarchical clustering of the differentially abundant proteins distinguished by *APOE* status following covariate adjustment using the Dream function. (C) (Upper) Number of nominally significant up‐ and downregulated proteins. (Lower) Circos plot mapping of protein family types measured in differentially abundant proteins between *APOE3* and *APOE4* subjects. Individual color‐coded labels indicate protein category types. (D) Top 10 differentially abundant proteins ranked by log_2_ fold‐change between *APOE3* and *APOE4* subjects following the Dream function. (E) Sample top proteins plotted using residualized Dream expression values. *P*‐values were calculated with Dream.

To evaluate downstream differences in plasma protein abundance associated with *APOE4* expression, we performed a linear mixed effects regression analysis on amyloid‐positive, homozygous *APOE3* and *APOE4* subjects using the Dream^34^ function and controlled for sources of biological and technical variability (Fig. S1A,B). We identified 80 *APOE4*‐associated plasma proteins that were up‐ or downregulated (Fig. 2A) with nominal statistical significance (*P* < 0.05). Unsupervised hierarchical clustering revealed distinct separation of plasma protein abundance between *APOE3* and *APOE4* subjects (Fig. 2B). Sizeable proportions of the 39 elevated proteins and 41 decreased proteins belonged to diverse protein categories (Fig. 2C), as visualized using protein‐type identification analysis. The top 10 differentially abundant proteins included NME2, UNC5D, EFNB2, CFP, and PPBP, as well as SLAMF7, CRP, AURKA, FUT5, and PAK6, which were up‐ and downregulated, respectively, in *APOE4* relative to *APOE3* subjects (Fig. 2D). Interestingly, NME2, which appears elevated in *APOE4* subjects, is an important regulator of the calcium‐activated K+ channel, K_Ca_3.1. These channels play a role in mediating CD4+ T‐cell activation,^38^ a process that may be dysregulated in AD.^39^, ^40^ SLAMF7 was the top downregulated plasma protein associated with *APOE4* (Fig. 2E). This protein has been implicated as a key regulator of adaptive immunity,^41^ a process that may regulate AD pathogenesis.^42^ Together, these data indicate that *APOE* allelic variation can alter plasma proteins previously implicated in AD prior to symptom onset.

### *APOE4* is associated with altered pathways in plasma associated with inflammation and CNS function

**Figure 3:**
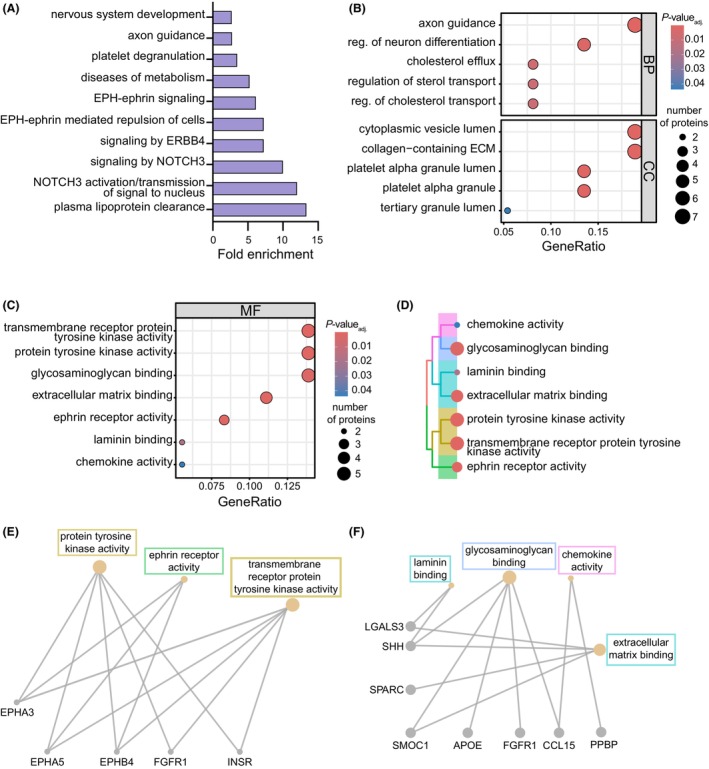
*APOE4* expression alters plasma protein pathways and associated networks. (A) Pathway enrichment analysis using the Reactome database from DAVID. The top 10 pathways using EASE scores (*P* < 0.05) are shown with corresponding fold‐enrichment values, calculated as proportion of input proteins and total number of proteins associated with that pathway. (B and C) Gene ontology over‐representation analysis was performed using *APOE4*‐associated proteins for selected biological processes, cellular components, and molecular functions (MF). Selected MF terms visualized by color for significance (adjusted *P*‐values (FDR < 0.05)) and size for the number of represented proteins. (D) MF terms were further analyzed by a hierarchical treeplot, where distance on the plot indicates similarity, color indicates significance, and size indicates the number of proteins. (E and F) Visualization of these MF terms by gene network plots was used for further investigation.

To evaluate whether plasma proteomic pathways are dysregulated according to *APOE4* status, we submitted differentially abundant plasma proteins to Ingenuity Pathway Analysis (IPA), revealing several significant pathways (FDR < 0.05) associated with immune function, including “STAT3” and “IL‐15 production”, as well as predicted changes in “atherosclerosis” pathways (Fig. S2A). Surprisingly, brain‐specific pathways were also identified from *APOE4*‐driven plasma protein changes, including changes in “synaptogenesis” and “axonal guidance.” We also accessed the Reactome pathway database through DAVID, which is preferentially modeled using biological reaction data,^43^ to analyze altered *APOE4*‐associated pathways using EASE scores (*P* < 0.05). This analysis highlighted expected changes^44^ in “plasma lipoprotein clearance” and “diseases of metabolism” (Fig. 3A). Interestingly, we identified brain‐specific pathways, including “axon guidance” and “nervous system development,” reinforcing CNS‐related pathways identified earlier by IPA and suggesting that expression of *APOE4* may alter pathways within the periphery and CNS. We next sought to explore putative functional changes associated with *APOE4* in plasma by evaluating gene ontology terms enriched by *APOE4*‐associated plasma proteins by over‐representation analysis (Fig. 3B–C). Similar to pathway analyses, expected functions known to be associated with the *APOE‐ε4* allele, including efflux of cholesterol and its transport, were enriched in biological processes (Fig. 3B). Several biological processes and cellular components were similarly altered compared to IPA and Reactome analyses, including “axon guidance” and changes to “platelet granulation.” We also found consensus with “collagen‐containing ECM” identified from cellular components (Fig. 3B) and molecular function terms related to the extracellular matrix (ECM), including “ECM,” “laminin,” and “glycosaminoglycan binding” (Fig. 3C). Using the enriched terms in Fig. 3C, we next examined the underlying plasma proteins associated with these functions and others and their relatedness by constructing a hierarchical treeplot. This plot revealed relatedness between tyrosine kinase activity and ephrin signaling terms, as well as clustering among chemokine activity and ECM/laminin and glycosaminoglycan binding terms (Fig. 3D). We then created gene–concept network plots from the extracted proteins from these terms, which highlighted the overlap of proteins in “tyrosine kinase activity” and ephrin receptor activity (Fig. 3E) pathways, as well as those related to ECM, glycosaminoglycan binding, and chemokine activity (Fig. 3F). Together, our results suggest that expression of *APOE4* is associated with plasma proteins linked to altered immune, ECM, and CNS‐linked pathways, among others.

### *APOE4* is associated with co‐regulatory protein expression changes

To examine how plasma proteins are co‐expressed and co‐regulated in the *APOE4* plasma proteome relative to *APOE3*, we used a weighted gene co‐expression network analysis (WGCNA) (Fig. S2B–F), which revealed a module (cyan) that was positively correlated with *APOE4* status (*P* = 3.22 × 10^−2^) (Fig. S3A,B). We extracted proteins from the cyan module and analyzed the mapping of several hub proteins (Fig. S3C), which were found to be related to ECM function (SPARC, THBS1, and PDGFB) and immune/inflammatory responses (PF4 and PPBP). To identify putative functional changes of the cyan module broadly, we performed over‐representation analysis for gene ontology terms on cyan module proteins (Fig. S3D). We identified biological processes related to immune cell movement and migration (“leukocyte migration” and “leukocyte chemotaxis”), while cellular components and molecular functions were related to the ECM (“collagen‐containing ECM,” “basement membrane,” “glycosaminoglycan binding,” “heparin binding,” and “collagen binding”). To investigate possible interactions among *APOE4*‐associated proteins, we next constructed physical and functional protein networks from the associated proteins using STRING (Fig. S4), revealing significant protein–protein interaction enrichment (*P* = 1.25 × 10^−7^). As expected, several proteins related to tyrosine kinase activity show strong putative interactions with one another (EPHA3, EPHB4, EFNB2, and EPHA5). We also found EGF as a potential hub protein with the greatest number of connections to other *APOE4*‐associated proteins within the network, including with other growth factors (FGFR1, PDGFRA, and NOG). Many of its connections were with proteins linked to pathways we previously highlighted, including immune function (LGALS3, SHH, and APOE), ECM (LGALS3, SPARC, and SHH), and metabolism (INSR, LGALS3, NOTCH3, and APOE). Together, these data suggest that altered immune function and ECM regulation may be critical mediators of broad *APOE4*‐mediated regulatory changes within the plasma proteome, and putative interactions of proteins underlying these pathways may indicate functional interconnectedness associated with *APOE4*.

### Impact of brain amyloid on plasma proteins in *APOE3* subjects

**Figure 4:**
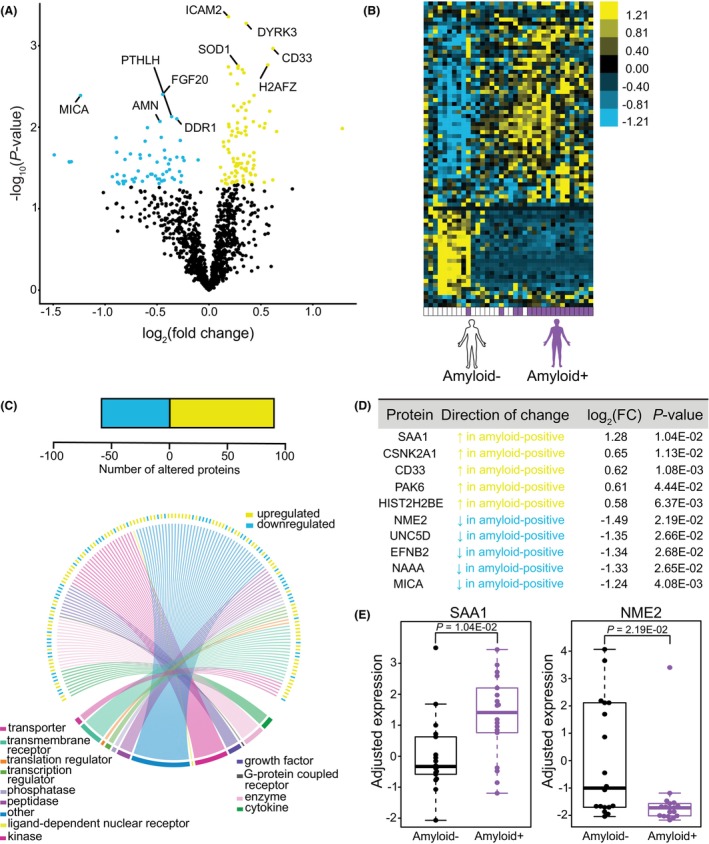
Plasma proteome features in amyloid‐positive and amyloid‐negative, *APOE3* subjects without dementia. (A) Volcano plot showing the fold‐change of proteins (log_2_ scale) between amyloid‐positive and amyloid‐negative subjects and corresponding nominal *P*‐values (−log_10_ scale) with upregulated proteins highlighted in yellow and downregulated proteins highlighted in blue. (B) Heat map showing unsupervised hierarchical clustering of the differentially abundant proteins distinguished by brain amyloid status following covariate adjustment using the Dream function. (C) (Upper) Number of nominally significant up‐ and downregulated proteins. (Lower) Circos plot mapping of protein family types measured in differentially abundant proteins between *APOE3* subjects who are amyloid‐positive or amyloid‐negative. Individual color‐coded labels indicate protein category types. (D) Top 10 differentially abundant proteins ranked by log_2_ fold‐change between *APOE3* amyloid‐positive and amyloid‐negative subjects following the Dream function. (E) Sample top proteins plotted using residualized Dream expression values. *P*‐values were calculated with Dream.

We next sought to examine whether brain amyloid positivity itself was associated with altered plasma protein abundance independent of *APOE4* status, which might imply disease‐specific peripheral dysregulation. We examined relative protein abundance by SOMAscan from 36 *APOE3* subjects, 18 of whom were categorized as amyloid‐positive, while the other 18 were amyloid‐negative (Fig. 1B; Fig. S1C), based on a published fluid biomarker threshold for the presence of brain amyloid used to identify samples for our cohort.^31^ *APOE4* subjects were not included based on insufficient numbers of amyloid‐negative *APOE4* subjects available in the cohort for comparison. Following adjustment of covariates, we identified nominally significant upregulated or downregulated proteins associated with the presence of brain amyloid (Fig. 4A). Unsupervised hierarchical clustering revealed a distinct plasma protein profile in subjects according to the presence of probable brain amyloid (Fig. 4B). Notably, we found 91 proteins that appeared to be elevated, and 61 proteins were decreased in amyloid‐positive relative to amyloid‐negative subjects (Fig. 4C), all of which were well represented across protein classes. The top 10 differentially abundant proteins distinguished by presence of brain amyloid in *APOE3* subjects were SAA1, CSNK2A1, CD33, PAK6, HIST2H2BE, as well as NME2, UNC5D, EFNB2, NAAA, and MICA, which were up‐ and downregulated, respectively (Fig. 4D,E). Notably, CD33, a top AD risk gene linked to microglial function that was identified through genome‐wide association studies,^45^ was upregulated in plasma in amyloid‐positive subjects (Fig. 4D). We also found that SAA1, a protein known to be highly abundant in AD cases versus controls,^46^, ^47^ was the top elevated plasma protein in amyloid‐positive versus amyloid‐negative *APOE3* subjects, with NME2 as the top downregulated protein in subjects with probable amyloid (Fig. 4E).

### Brain amyloid is associated with altered pathways in plasma of *APOE3* subjects

**Figure 5:**
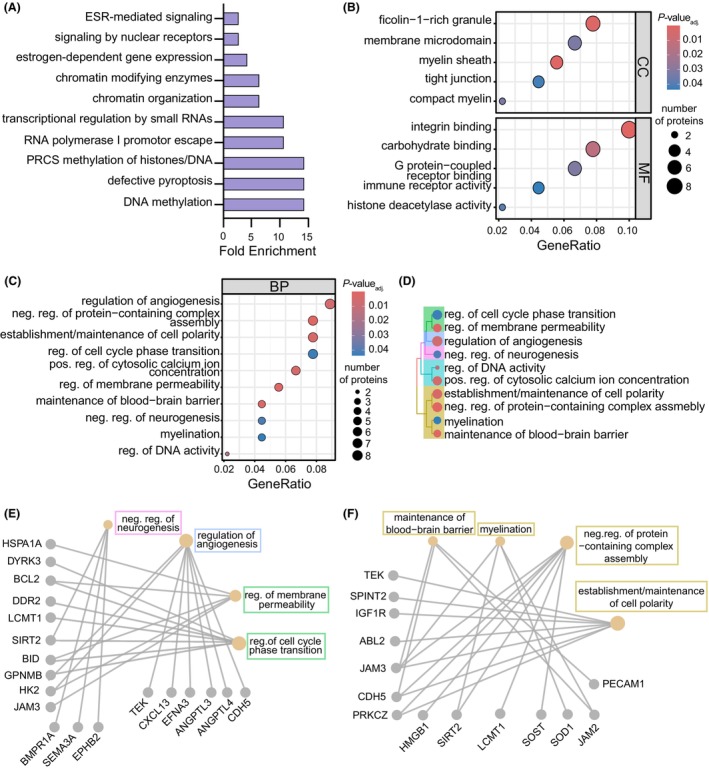
Pathway and network annotations differ between amyloid‐positive and amyloid‐negative, *APOE3* subjects. (A) Pathway enrichment analysis using the Reactome database from DAVID. Top 10 pathways using EASE scores (*P* < 0.05) were shown with corresponding fold‐enrichment values, calculated as proportion of input proteins and total number of proteins associated with that pathway. (B and C) Gene ontology over‐representation analysis was performed using amyloid‐associated proteins for selected cellular components, molecular functions, and biological processes (BP). Selected BP terms visualized by color for significance (adjusted *P*‐values (FDR < 0.05)) and size for the number of represented proteins. (D) BP terms were further analyzed by a hierarchical treeplot where distance on the plot indicates similarity, color indicates significance, and size indicates the number of proteins. (E and F) Visualization of these BP terms by gene network plots was used for further investigation.

To examine pathways in the plasma proteome disrupted in subjects with probable brain amyloid, we submitted differentially abundant plasma proteins associated with amyloid positivity to IPA (Fig. S5A). Pathways related to altered immune function and inflammation observed in AD subjects^48^ were identified, including “immunogenic cell death,” “leukocyte extravasation,” and “IL‐15 production.” Further probing of pathways that may be altered according to brain amyloid status was conducted using the Reactome pathway database through DAVID (Fig. 5A), which highlighted pathways related to “DNA methylation” and “chromatin organization.” Additionally, a “defective pyroptosis” pathway was identified, possibly indicating processes linked to increased cell death.^49^ We further examined enriched pathways using gene ontology for over‐representation analysis, which highlighted changes in cellular component and molecular function terms related to “tight junctions,” “integrin binding,” and “immune receptor activity” (Fig. 5B). We also identified consensus between our pathway analyses and terms for biological processes and cellular components, including “histone deacetylase activity” and “regulation of DNA activity” (Fig. 5B,C). Notably, there were also several CNS‐related biological processes associated with the plasma proteomic differences linked to brain amyloid, including “myelination,” “negative regulation of neurogenesis,” “regulation of angiogenesis,” and “maintenance of blood–brain barrier” (Fig. 5C). Given links in AD to neurogenesis^50^ and blood–brain barrier (BBB) disruption,^51^ we explored the plasma proteins underlying these associations and the related terms through a hierarchical treeplot (Fig. 5D). “Negative regulation of neurogenesis” was related to cellular functions (“regulation of cell cycle phase transition” and “regulation of membrane permeability”) and “regulation of angiogenesis”, while “maintenance of blood–brain barrier” was closely associated with “myelination,” “establishment/maintenance of cell polarity,” and “negative regulation of protein‐containing complex assembly” terms (Fig. 5D). As expected, overlapping plasma proteins for “negative regulation of neurogenesis” identified by gene‐network construction were related to cell cycle phase transition (SIRT2) (Fig. 5E). Interestingly, overlapping plasma proteins between “maintenance of blood–brain barrier” and “myelination” terms were also identified, including JAM2 and JAM3 (Fig. 5F). Our results point to plasma proteomic changes associated with brain amyloid that are linked to CNS functions, including regulation of neurogenesis and of angiogenesis, BBB maintenance, and myelination, in subjects without dementia.

To further understand how plasma proteins altered in subjects with brain amyloid are co‐regulated, we employed WGCNA (Fig. S5B–F) and identified the brown module (*P* = 6.87 × 10^−3^) (Fig. S6A,B), whose mapped hub proteins, PECAM1, FCN1, CDH12, MMP17, and DDR2 (Fig. S6C) are predominantly related to immunity/inflammation, blood–brain barrier, matrix/tissue remodeling, and adhesion biology. To examine the relationships of these proteins with specific functions, we used over‐representation analysis for gene ontology terms (Fig. S6D), which highlighted molecular functions related to matrix remodeling (“heparin binding” and “glycosaminoglycan binding”), as well as many related molecular functions and cellular components related to inflammation and overall immune function (“chemokine activity,” “cytokine binding,” “azurophil granule lumen,” “ficolin‐1‐rich granule,” and “immune receptor activity”). We next used STRING to characterize possible interactions among amyloid‐associated proteins (Fig. S7), revealing a significant protein–protein interaction enrichment (*P* = 2.18 × 10^−11^). While many amyloid‐associated proteins appeared to be interconnected, HGF showed the highest number of these interactions, and several of these proteins were related to highlighted pathways, including inflammation (HMGB1, TNFAIP6, TNFRSF1A, AREG, and SAA1), cell–cell adhesion (PECAM1, CDH5, and CCN2), ECM (TNFAIP6 and CCN2), and apoptosis/cell death (BCL2, TNFRSF1A, and RPS27A), suggesting shared mechanisms among these functions. Together, our results suggest that harboring brain amyloid is associated with distinct changes in the plasma proteome that may be associated with altered immune, vascular, and remodeling processes relevant to CNS function.

## Discussion

Given growing recognition of the need to understand how peripheral dysregulation reflects or contributes to neurodegenerative diseases, we sought to characterize how variation in *APOE* genotype alters the plasma proteome in elderly subjects without dementia. Variance partition analysis shows a large source of variance from unreported factors (Fig. 1E), which may include pre‐analytical technical variation, lifestyle differences, comorbidities, use of prescription medications, differences in weight and cardiac health (blood pressure and pulse), among others. Given that presence of amyloid was a significant source of variation, we compared plasma proteins in amyloid‐positive *APOE3* subjects to amyloid‐positive subjects expressing the high‐risk *APOE‐ε4* alleles (Fig. S1B). We found that *APOE* genotype influences levels of various plasma proteins, consistent with earlier reports in *APOE4*‐carriers versus non‐carriers for a subset of proteins.^19^ We found *APOE*‐related alterations in proteins related to immune function, including in NME2 and SLAMF7, which are implicated in downstream activation of CD4+ T‐cells and adaptive immunity, respectively. Accumulating evidence has demonstrated a significant relationship between *APOE* and innate immune function in AD^52^, and evidence for a relationship between *APOE4* and adaptive immunity is emerging.^42^, ^53^ CRP, which assumes both innate and adaptive immune roles, is decreased in *APOE4* relative to *APOE3* subjects in CSF^54^ and serum.^55^, ^56^ Chronically low levels of CRP were also associated with accelerated AD onset.^56^ These studies are consistent with our observation that CRP levels appear to be decreased in *APOE4* subjects without dementia (Fig. 2D). From pathway analyses, we found that *APOE4*‐associated plasma changes were consistent with perturbations in functions related to apoE biology and ECM function (Fig. 3A–D), findings that were highlighted throughout gene ontology over‐representation analyses. *APOE4*‐associated alterations in regulation of sterol and cholesterol transport, are consistent with the canonical function of apoE protein and the impact of its polymorphisms.^44^ We also noted a modest *APOE4*‐associated enrichment in pathways related to metabolic dysfunction, a phenotype that has been associated with AD risk^57^ and could be a link to cerebrovascular changes. A recent study examining plasma proteins in AD cases and controls reported changes related to lipid biosynthesis/immune response and ECM organization correlated with the *APOE4* allele.^58^ Moreover, a rare variant in *FN1*, the gene encoding the ECM component fibronectin‐1, was found to be protective for AD in *APOE4* subjects.^59^ Our work examining age‐associated human and mouse plasma proteins and their impact in blood–brain communication also implicate proteins that regulate the ECM,^3^, ^60^ and a relationship between *APOE4* and ECM function has been suggested in the brain.^61^, ^62^ Analysis of broad protein relationships by WGCNA or associations identified by STRING in our study revealed co‐regulation and interactions of *APOE4*‐associated proteins related to the ECM, (Figs. S3 and S4), but future work should validate *APOE4's* role in regulating peripheral ECM function. Consensus between pathway analyses and gene ontology terms for ephrin receptor activity was also identified in *APOE4* subjects (Fig. 3A,C), which, intriguingly, mirrors *APOE* isoform‐related differences in neuronal excitability in mice.^63^ As expected, plasma proteins associated with ephrin receptor activity were shared with tyrosine kinase activity terms (Fig. 3C–E). Recent work found that Axl, a receptor for tyrosine kinase, differs at baseline between *APOE4* and *APOE3* microglia,^64^ and tyrosine kinases have been suggested to modulate microglial migration, perhaps related to these differences.^65^ A previous study evaluating plasma protein changes in *APOE4* subjects identified unique “dementia‐associated proteins.”^66^ While only two of the six proteins identified were measured in our study, both proteins, IGFBP2 and F10, were not significant in our evaluation of *APOE4*‐associated plasma proteins in our cohort of elderly subjects without dementia, perhaps arguing that differences might be related to disease stage. A separate study following centenarians and their offspring identified 16 *APOE*‐associated serum proteins that were capable of predicting future disease susceptibility,^19^ further supporting that *APOE* alleles are associated with a perturbed circulating proteome in human subjects.

Using a biomarker‐based cutoff indicative of the presence of brain amyloid,^29^, ^30^ we examined how suspected brain amyloid affects the plasma proteome in *APOE3* subjects (Fig. 4; Fig. S1C). We found widespread changes in plasma protein abundance in subjects exhibiting brain amyloid biomarkers. One protein in particular, NME2, was upregulated in amyloid‐negative *APOE3* subjects (Fig. 4E), as well as in amyloid‐positive *APOE4* subjects (Fig. 2E), possibly suggesting differing regulation of NME2‐K_ca_3.1 function in these contexts. Regulation of Ca^2+^ signaling through K‐_ca_3.1 has been attributed to microglial activation,^67^ and its pharmacological blockade induces anti‐inflammatory effects in amyloid‐bearing mice,^68^ possibly linking changes in NME2 abundance to documented neuroinflammatory changes in *APOE4*‐carriers,^52^ though other functions include synthesis of nucleoside triphosphates, lipid membrane binding, and endocytic processes.^69^ Thus, it is possible that NME2 levels in these two groups reflect *APOE4*‐associated dysfunction in the setting of amyloid of a normally protective function in *APOE3*‐carriers. Plasma levels of SAA1 were found to be upregulated in amyloid‐positive subjects, which was supported by enrichment of gene ontology terms linked to inflammation. Other ontology‐based analyses in these groups highlighted changes in processes linked to AD pathogenesis, including the blood–brain barrier,^51^ programmed cell death by pyroptosis,^49^ and altered epigenetic mechanisms related to DNA and chromatin function.^70^ A study measuring plasma proteins in AD cases and controls highlighted some of these pathways along with non‐overlapping pathways,^18^ perhaps due to the more advanced ages and/or pathology in their cohort. Several other CNS‐associated processes identified in our study have been similarly linked to AD pathogenesis, including altered adult neurogenesis,^50^ and myelination.^71^ Protein co‐regulatory changes identified using WGCNA (Fig. S6) indicate broad but interconnected changes in functions related to immunity/inflammation, adhesion, and tissue remodeling. Comparing these pathways and functions related to protein co‐regulation in our dataset to previous work,^18^ there is consensus, including regulation of “endothelial cell migration,” changes in “cytokine binding,” and “G protein‐coupled receptor binding” (Fig. S6D). Shared pathways between the datasets may indicate broad phenotypes in the plasma proteome reflecting the long pathological process of AD across preclinical to symptomatic stages. Using established cut‐offs for CSF Aβ42 concentration to reflect probable presence of brain amyloid in research subjects has been a reliable tool,^72^ yet new markers and combinatorial approaches will likely improve the detection of brain amyloid in living subjects and subsequent proteomic studies. Previous work examined changes in the plasma proteome in AD cases and controls,^18^, ^73^ though few studies have explored the plasma proteome prior to disease onset.

Our study, while discovery‐oriented, uniquely highlights changes in the systemic environment in subjects without dementia according to *APOE* status and brain amyloid status. Several pathways or top *APOE4*‐ and amyloid‐associated proteins are consistent with previous work.^18^, ^20^, ^21^ Differences in cohort characteristics, including disease stage, age, demographics, *APOE* genotypes represented, and varying sample preparation and cohort size all may account for minimal overlap identified among recent proteomic analyses. Previous studies examining proteomic changes associated with the *APOE4* allele included subjects with AD or cognitive impairment^58^ or subjects who were carriers of the *APOE4* allele,^19^ for example. Additionally, without the inclusion of AD subjects in our study, amyloid‐associated proteins we identified should be further verified in longitudinal studies to explore whether these proteins relate to AD progression. A further limitation is our study's modest cohort size, a consequence of our strategy to limit samples to elderly, homozygous *APOE3* or *APOE4* subjects without dementia not represented in other work. Future efforts in larger cohorts with downstream validation may increase confidence in the identified pathways and specific proteins that may drive their function.

## Conflict of Interest

J.M.C. is listed as a co‐inventor on patents for treating aging‐associated conditions, including the use of young plasma administration (US10688130B2) or youth‐associated protein TIMP2 (US10617744B2), the latter of which is licensed to Alkahest, Inc.

## Author Contributions

S.M.P. performed data analysis and wrote the manuscript. J.M.C. collected data, designed and supervised the research, and wrote the manuscript. K.BP and T.R. assisted with data analysis and writing of the manuscript. All authors interpreted and discussed the results.

## Supporting information


Figure S1.
Figure S2.
Figure S3.
Figure S4.
Figure S5.
Figure S6.
Figure S7.

## Data Availability

The data underlying the described results are available in the published article from the GEO repository (GSE275392).
