# Supplementary material for: APOE genotype and brain amyloid are associated with changes in the plasma proteome in elderly subjects without dementia
Source: Ann Clin Transl Neurol. 2024 Dec 17;12(2):366–82. doi: 10.1002/acn3.52250 (PMC11822792; doi:10.1002/acn3.52250)
Supplement: Supplementary file 1 — Figure S1. Figure S2. Figure S3. Figure S4. Figure S5. Figure S6. Figure S7. [file ACN3-12-366-s001.pdf]

Supplementary Figure S1

A

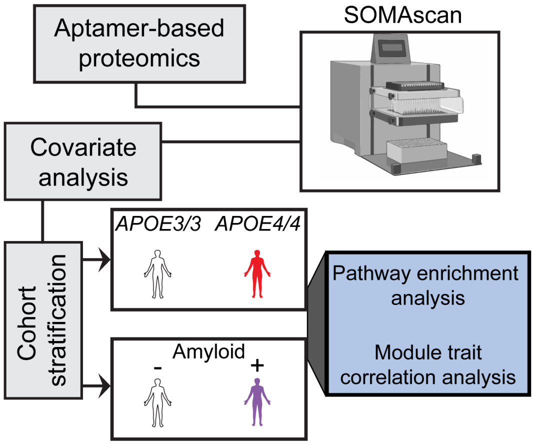

B

	APOE4-associated				
	Sex	*N*	Mean Age	MMSE Score	% CDR 0
APOE3/3	Male	12	71.26 (± 6.77)	28.83 (± 1.47)	100
	Female	6	64.00 (± 9.27)	29.67 (± 0.52)	100
APOE4/4	Male	6	68.40 (± 9.31)	28.50 (± 1.38)	100
	Female	11	66.46 (± 7.62)	29.27 (± 1.01)	100

C

	Amyloid-associated				
	Sex	*N*	Mean Age	MMSE Score	% CDR 0
Amyloid-negative	Male	9	66.12 (± 7.47)	28.89 (± 1.17)	100
	Female	9	69.88 (± 7.97)	29.44 (± 0.53)	100
Amyloid-positive	Male	12	71.26 (± 6.77)	28.83 (± 1.47)	100
	Female	6	64.00 (± 9.27)	29.67 (± 0.52)	100

Supplementary Figure S1.

Cohort stratification for downstream analyses in *APOE3* and *APOE4* subjects without dementia.

A. Schematic workflow describing cohort stratification following covariate analysis from plasma proteins measured by the SOMAscan assay. **B.** 35 male and female subjects homozygous for *APOE3* or *APOE4* and identified as amyloid-positive using a CSF Aβ42 cut-off were separately analyzed in "*APOE4*-associated" proteomic analyses. **C.** 36 homozygous *APOE3* male and female subjects were separately analyzed to identify brain "amyloid-associated" plasma proteins independent of the *APOE4* allele.

Supplementary Figure S2

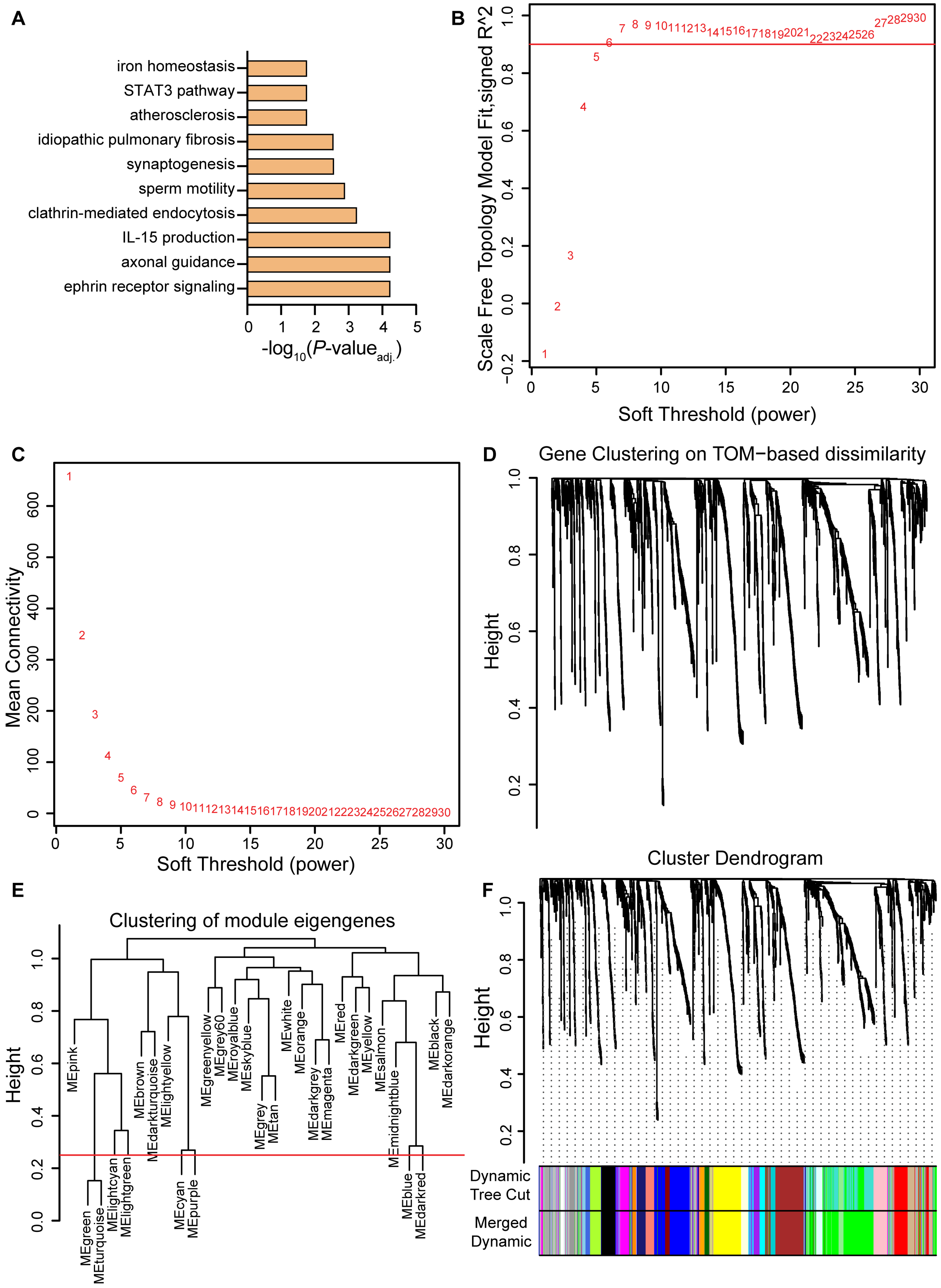

Supplementary Figure S2.

Pathway analysis and network construction for co-regulatory protein analysis between *APOE4* and *APOE3* subjects. A. Ingenuity Pathway Analysis for plasma proteins upregulated in *APOE4* relative to *APOE3* subjects. Significance is represented by the adjusted (FDR < 0.05) -log(*P-value*) for the top 10 pathways. **B.** Network parameters were selected for soft thresholding powers. Scale-free topology is attained above the red line. **C.** Mean connectivity for further soft thresholding analysis was selected as values approach 0. **D.** Gene clustering for dissimilarity by topological overlap. **E.** Clustering of module eigengenes to identify overlapping modules was selected using a merge height of 0.25 (red line). **F.** Cluster dendrogram of topological overlap with colored bars to indicate module assignments. Dynamic tree cut shows the unmerged module assignments related to the merged dynamic for the final module colors.

Supplementary Figure S3

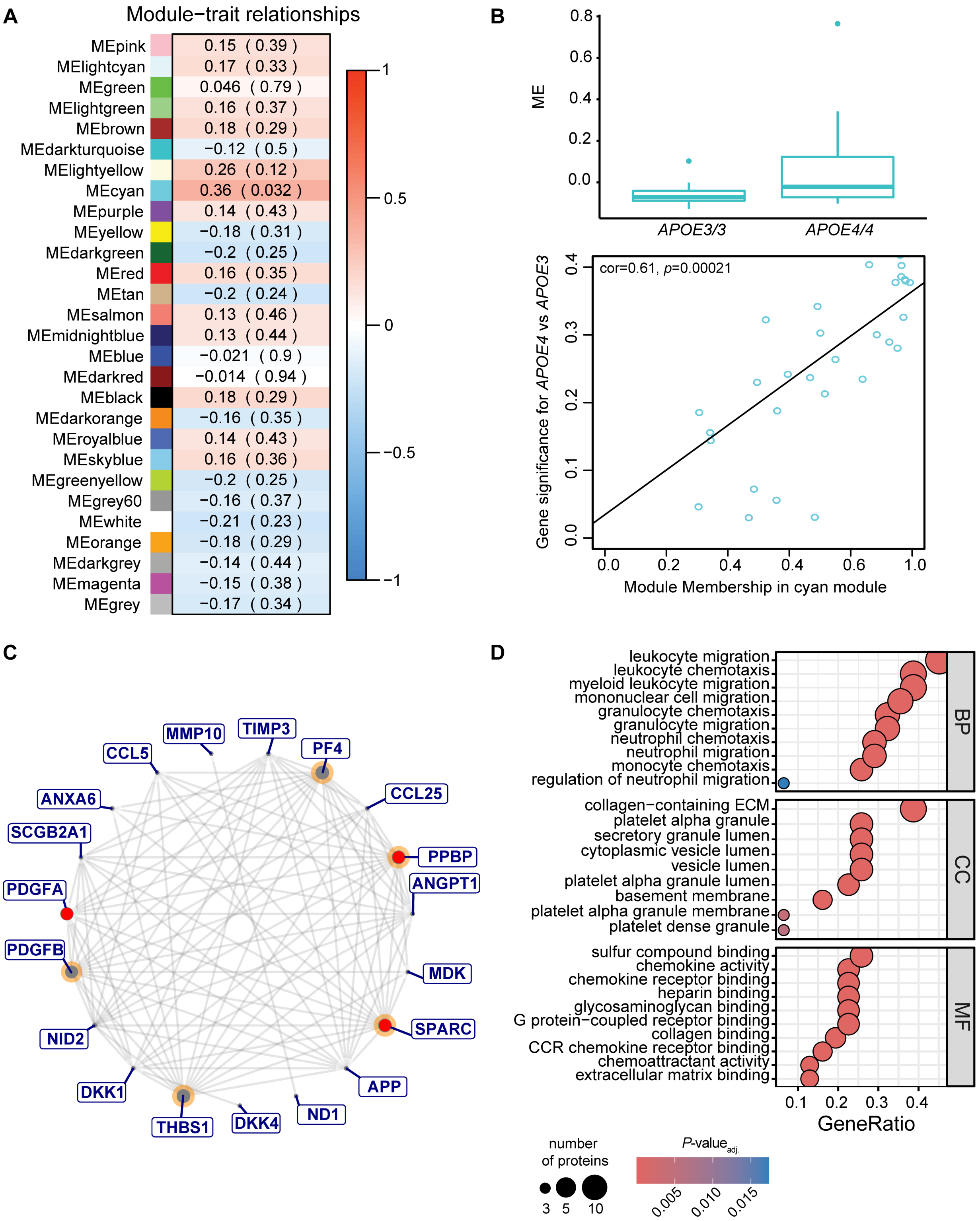

Supplementary Figure S3.

WGCNA module selection for co-regulatory changes between *APOE4* and *APOE3* subjects. **A.** Construction of the module-trait relationships plot for all module assignments (color labels), in addition to the module membership values and the respective module significance in parentheses at alpha = 0.05. Selection of modules for further analysis is based on significance. **B.** Bar graph demonstrating module eigengene differences for the cyan module between *APOE4* and *APOE3* subjects. Scatterplot indicates the correlation among individual module eigen values and comparison between *APOE* status. **C.** Extraction of proteins from the cyan module were visualized for protein-protein interactions by node and edge connectivity. Identification of hub genes (orange) was made by assessing module membership values, while upregulated proteins identified previously in Dream are highlighted in red. **D.** Over-representation analysis for gene ontology was performed using *APOE4*-associated proteins for select biological processes (BP), cellular components (CC), and molecular functions (MF).

Supplementary Figure S4

A

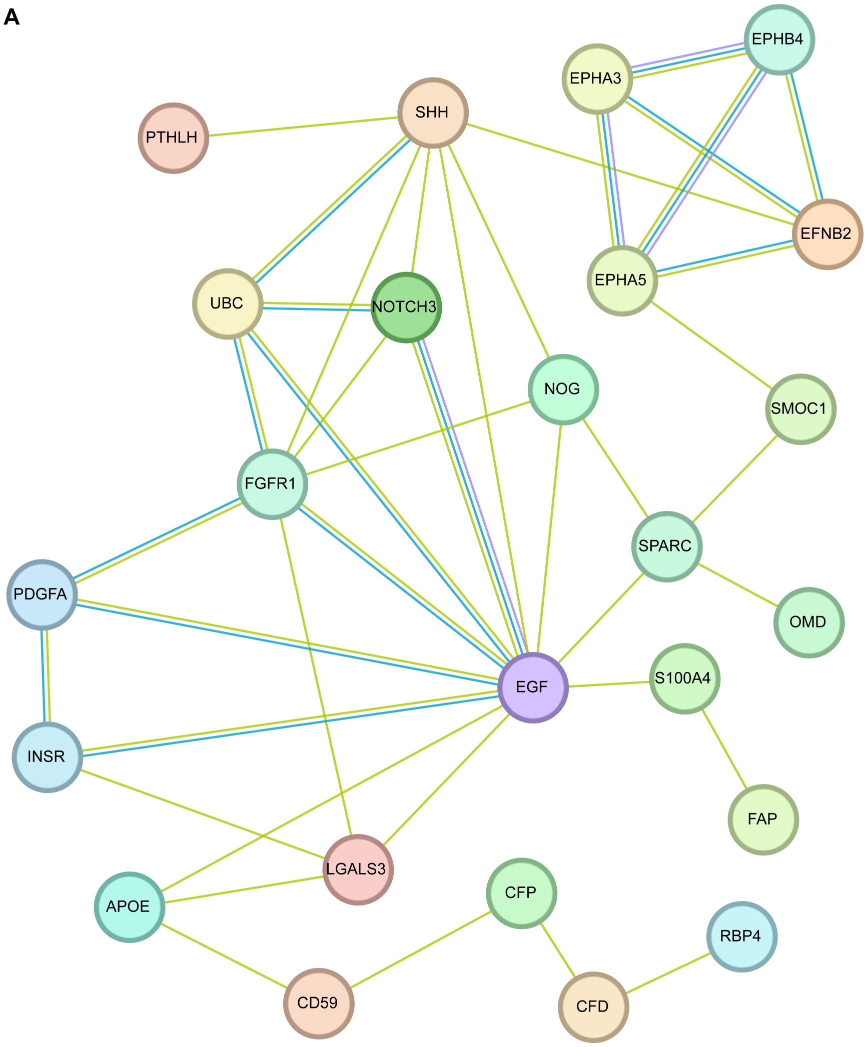

Supplementary Figure S4

Protein-protein interaction network for plasma proteins identified between *APOE4* and *APOE3* subjects

A. Functional and physical protein association network for *APOE4*-associated plasma proteins from **Figure 3** using STRING (Search Tool for the Retrieval of Interacting Genes/Proteins). Network edges with a minimum confidence interaction of 0.400 were used from “text mining” and “database” entries.

Supplementary Figure S5

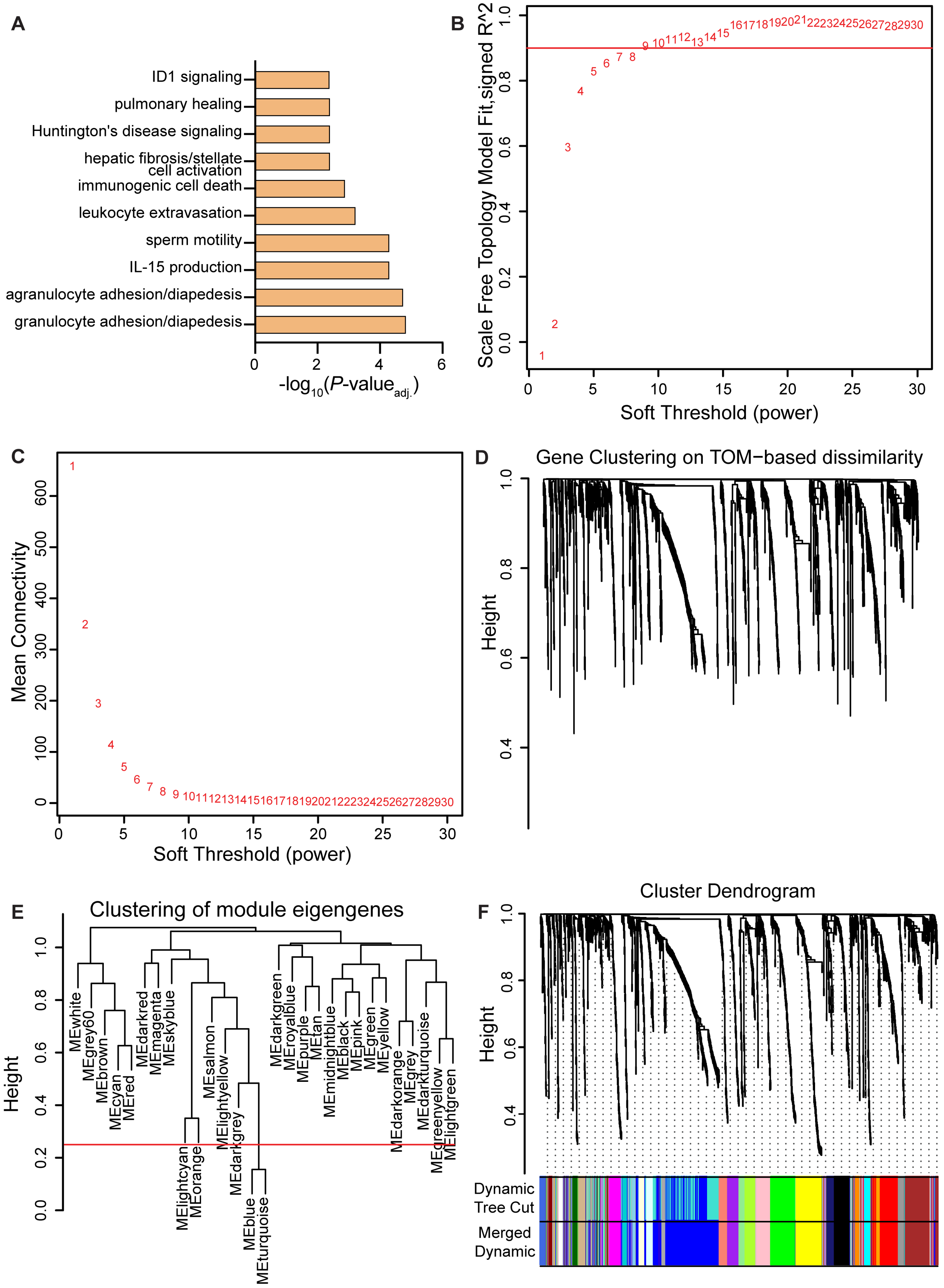

Supplementary Figure S5.

Pathway analysis and network construction for co-regulatory protein analysis between amyloid-positive and amyloid-negative, *APOE3* subjects. A. Ingenuity Pathway Analysis (IPA) for plasma proteins upregulated in subjects with fluid biomarkers indicative of brain amyloid positivity. Significance is represented by the adjusted (FDR < 0.05) -log(p-value) for the top 10 pathways. **B.** Network parameters were selected for soft thresholding powers. Scale-free topology is attained above the red line. **C.** Mean connectivity for further soft thresholding analysis was selected as values approach 0. **D.** Gene clustering for dissimilarity by topological overlap. **E.** Clustering of module eigengenes to identify overlapping modules was selected using a merge height of 0.25 (red line). **F.** Cluster dendrogram of topological overlap with colored bars to indicate module assignments. Dynamic tree cut shows the unmerged module assignments related to the merged dynamic for the final module colors.

Supplementary Figure S6

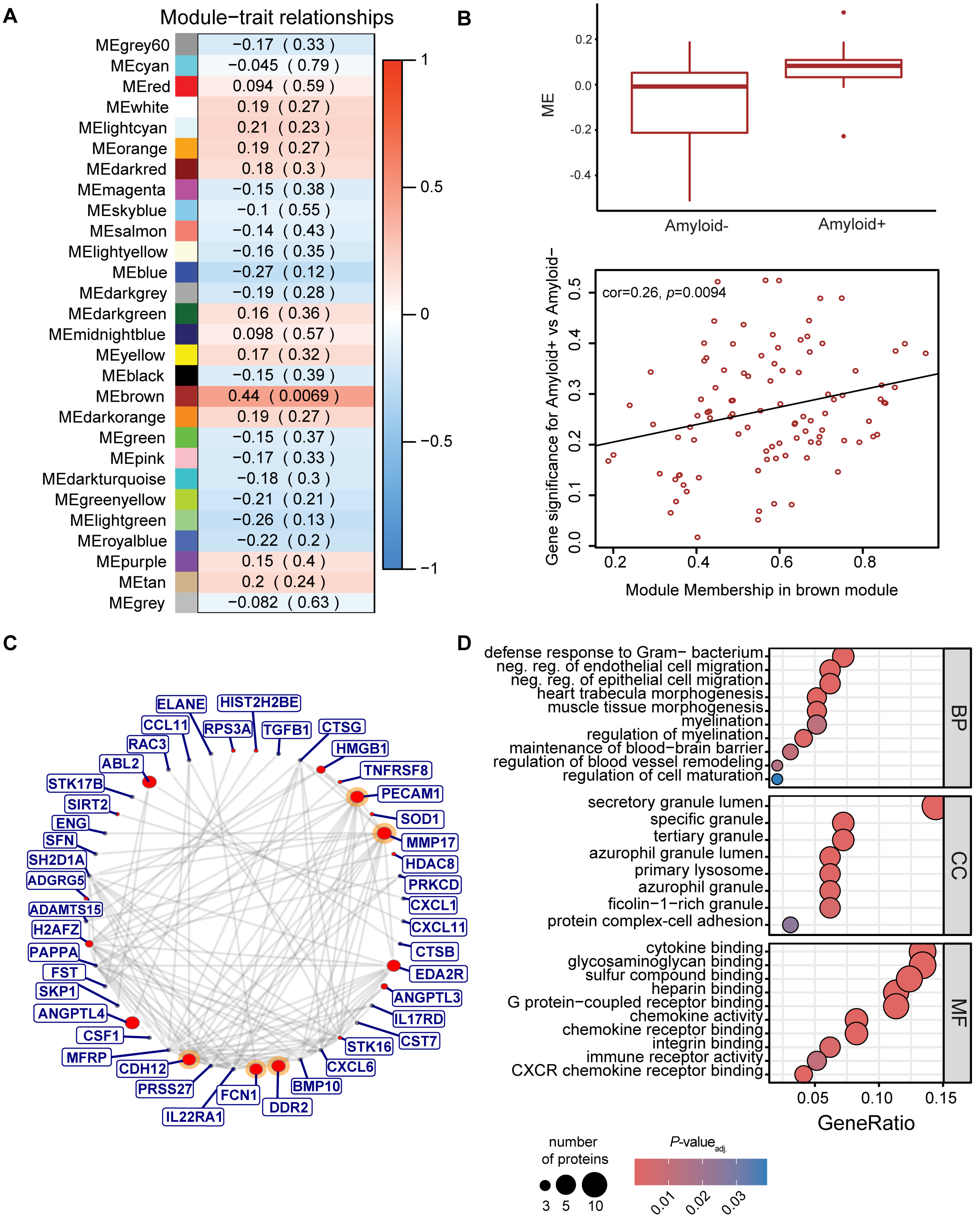

Supplementary Figure S6.

WGCNA module selection for co-regulatory changes between amyloid-positive and amyloid-negative, *APOE3* subjects without dementia. A. Construction of the module-trait relationships plot for all module assignments (color labels) in addition to the module membership values and the respective module significance in parentheses at alpha = 0.05. **B.** Bar graph demonstrating module eigengene differences for the brown module between amyloid-positive and amyloid-negative subjects. Scatterplot indicates the correlation among individual module eigen values and amyloid-positivity. **C.** Extraction of proteins from the brown module were visualized for protein-protein interactions by node and edge connectivity. Identification of hub genes (orange) was done by assessing the module membership values, while upregulated proteins identified previously in Dream are highlighted in red. **D.** Over-representation analysis for gene ontology terms was performed using upregulated (amyloid-positive) proteins for select biological processes (BP), cellular components (CC), and molecular functions (MF).

Supplementary Figure S7

A

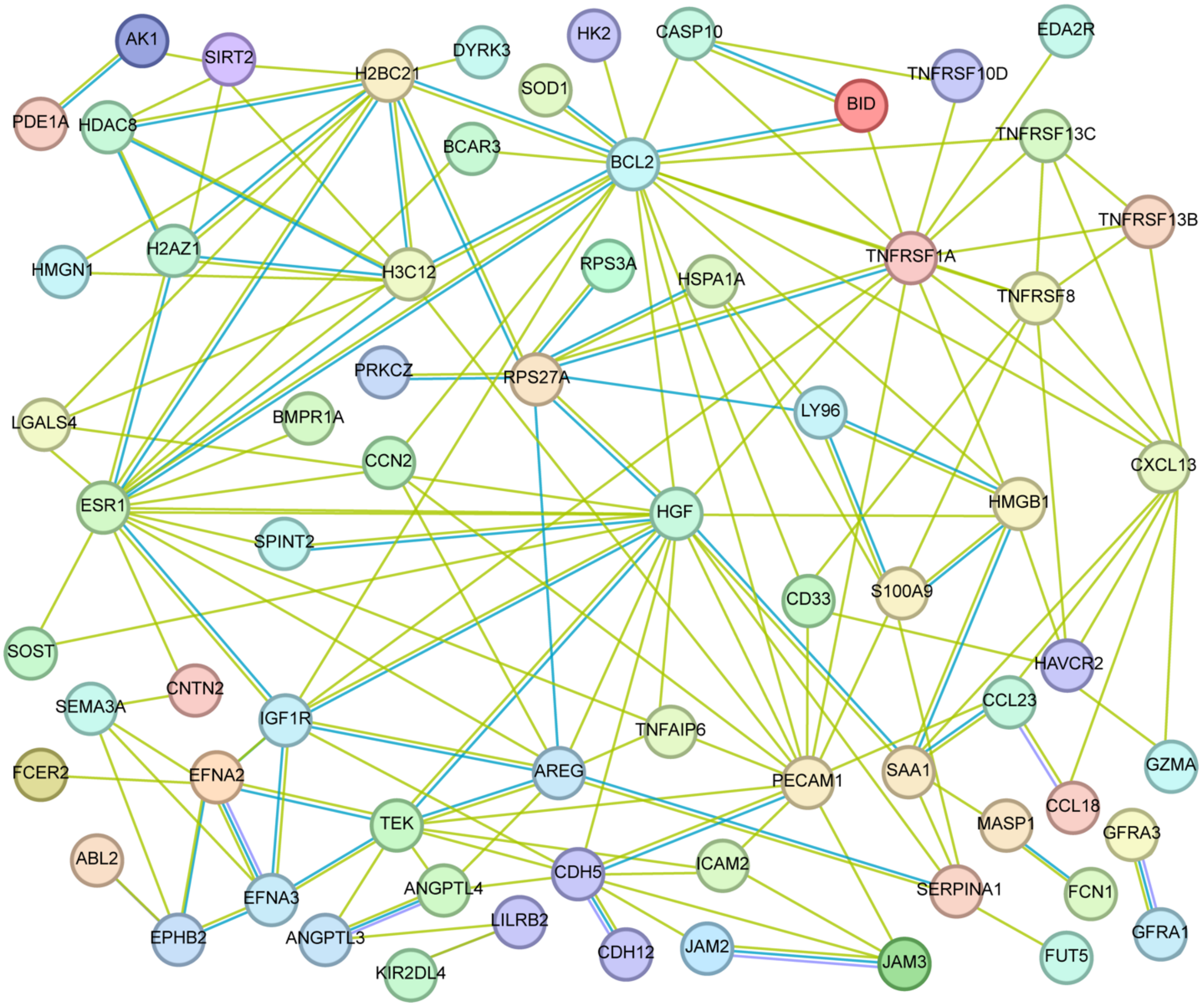

Supplementary Figure S7

Protein-protein interaction network from plasma of *APOE3* subjects who are amyloid-positive or amyloid-negative

A. Functional and physical protein association network for amyloid-positive plasma proteins from **Figure 5** using STRING (Search Tool for the Retrieval of Interacting Genes/Proteins). Network edges with a minimum confidence interaction of 0.400 were used from “text mining” and “database” entries.
